# Determination of the 90% effective dose of propofol combined with oliceridine or fentanyl for inhibiting the insertion response in upper gastrointestinal endoscopy: a dose-finding trial

**DOI:** 10.3389/fmed.2026.1813849

**Published:** 2026-05-20

**Authors:** Fangsheng Xu, Yuanyuan Cui, Ye Huang, Jian Tang, Yuan Xu, Mingcong Wang, Mengqi Liu, Kehui Qu, Jianfeng Pu, Meifang Wang, Qian Wang

**Affiliations:** 1 Department of Anesthesiology, Changshu No. 2 People’s Hospital, Changshu, China; 2 Department of Anesthesiology, Changshu Hospital Affiliated to Nanjing University of Chinese Medicine, Changshu, China

**Keywords:** biased coin design, ED_90_, fentanyl, oliceridine, propofol, upper gastrointestinal endoscopy

## Abstract

**Background:**

Propofol combined with opioids is commonly used for procedural sedation, enhancing patient tolerance and reducing the incidence of adverse events. This study aimed to determine the 90% effective dose (ED_90_) of propofol combined with fentanyl or oliceridine to inhibit the gastroscopy insertion response.

**Methods:**

This randomized, double-blind, dose-finding trial enrolled patients undergoing painless upper gastrointestinal endoscopy. Participants were assigned to receive propofol combined with fentanyl or oliceridine. A biased coin up-and-down sequential design was used to estimate the ED_90_ of propofol for inhibiting the gastroscopy insertion response. The primary outcome was the ED_90_ and 95% confidence intervals (CIs) for propofol in combination with either fentanyl or oliceridine for inhibiting the gastroscopy insertion response.

**Results:**

A total of 115 patients were included in the final analysis (59 in Group F and 56 in Group O). The ED_90_ of propofol required to suppress the gastroscopy insertion response was 1.942 (1.709–2.494) mg/kg in Group F and 1.871 (1.620–2.519) mg/kg in Group O. There were no significant between-group differences in the ED_90_ or in the incidence of adverse events (*P* > 0.05).

**Conclusion:**

The ED_90_ of propofol for suppressing gastroscopy insertion responses was 1.942 mg/kg (95% CI, 1.709–2.494) with fentanyl and 1.871 mg/kg (95% CI, 1.620–2.519) with oliceridine. The substantial overlap of the confidence intervals suggests comparable propofol requirements for the two opioid adjuncts under the study conditions. These findings provide quantitative reference data to guide individualized initial propofol dosing during upper gastrointestinal endoscopy.

**Clinical Trial Registration:**

https://www.chictr.org.cn/, identifier ChiCTR2500096971.

## Introduction

Upper gastrointestinal endoscopy (UGIE) is widely used for the diagnosis and treatment of upper gastrointestinal diseases (1). However, endoscope insertion often provokes gag reflexes, retching, coughing, and involuntary movements, which may compromise procedural quality and increase patient discomfort (2). Therefore, adequate sedation is essential to ensure patient comfort, procedural safety, and optimal endoscopic conditions.

Propofol is commonly used for sedation during UGIE because of its rapid onset and quick recovery profile. Nevertheless, its narrow therapeutic window and lack of intrinsic analgesic properties often necessitate higher doses to suppress insertion responses, increasing the risk of dose-dependent adverse effects such as respiratory depression and hypotension (3, 4). To enhance sedation quality and reduce propofol requirements, opioids are commonly added to propofol (5). Fentanyl, a short-acting μ-opioid receptor agonist, is one of the most frequently used opioid adjuncts during painless gastroscopy (6). Although fentanyl-propofol combinations are commonly titrated in clinical practice, the propofol dose required to reliably suppress gastroscopy insertion responses when combined with fentanyl has not been well defined (7). Estimating the effective dose of propofol for specific procedural endpoints may provide useful benchmark information for clinical dosing (8).

Oliceridine is a novel μ-opioid receptor agonist with G protein-biased signaling properties, designed to provide effective analgesia while potentially reducing opioid-related adverse effects (9). Emerging evidence suggests that oliceridine may be associated with a lower incidence of respiratory depression and hemodynamic instability compared with traditional opioids in procedural sedation (10). However, limited data are available regarding propofol dose requirements when oliceridine is used as an adjunct during UGIE.

Although opioid-propofol combinations are widely used for sedation during upper gastrointestinal endoscopy, quantitative characterization of propofol dose requirements across different opioid adjuncts remains limited. This study aimed to determine the 90% effective dose (ED_90_) of propofol when combined with fentanyl or oliceridine for suppressing gastroscopy insertion responses, providing comparative dose-finding reference data for clinical sedation protocols.

## Materials and methods

### Trial design

This study was approved by the Ethics Committee of Changshu No. 2 People’s Hospital (2024-KY-Y05) and registered with the Chinese Clinical Trial Registry (ChiCTR2500096971). All participants provided written informed consent prior to enrollment and were informed of their right to withdraw from the study at any time. This study was designed as a single-center, randomized, double-blind, dose-finding trial using a biased coin up-and-down sequential allocation method and was conducted in accordance with the Declaration of Helsinki.

### Participants

We aimed to enroll 120 patients scheduled for painless upper gastrointestinal endoscopy with procedural sedation at Changshu No. 2 People’s Hospital between February and October 2025. Inclusion criteria were: age 18–65 years, American Society of Anesthesiologists (ASA) physical status I-III, body mass index (BMI) between 18 and 30 kg/m^2^, and successful completion of a preoperative outpatient anesthesia evaluation. Patients were excluded if they had a history of esophageal or gastrointestinal surgery, documented arrhythmias, long-term use of opioids or sedatives, known allergies to anesthetic agents, requested withdrawal from the study, or if the procedure was converted to routine gastroscopy without sedation.

### Randomization and blinding

A block randomization design was employed, with random sequences generated using an online tool and block sizes of 4 or 8. Eligible participants were randomly assigned in a 1:1 ratio to receive either propofol combined with fentanyl (Group F) or propofol combined with oliceridine (Group O). The allocation sequence was sealed in opaque envelopes, and a trained study coordinator opened the envelope and prepared the corresponding medication just before each procedure. Anesthesiologists performed the anesthetic procedures, while anesthesia nurses were responsible for data collection. Throughout the trial, patients and their guardians, anesthesiologists, anesthesia nurses, endoscopists, and data analysts were blinded to group allocation. Study team members were not allowed to access allocation information until the completion of the trial, unless unblinding was required due to a serious clinical complication or unforeseen emergency.

### Study protocol

Patients fasted for at least 8 h from solids and 2 h from liquids prior to the procedure. No premedication was administered before induction. Upon entering the operating room, a peripheral intravenous catheter was inserted, and 0.9% sodium chloride was initiated at a maintenance rate. Standard monitoring included pulse oxygen saturation (SpO2), heart rate (HR), electrocardiogram (ECG), and non-invasive blood pressure (NIBP). Supplemental oxygen was administered via nasal cannula at a flow rate of 5 L/min.

Patients were placed in the left lateral decubitus position, and noninvasive blood pressure was monitored on the right upper arm. According to group allocation, patients in Group O received oliceridine 20 μg/kg (2 mL: 2 mg; Jiangsu Nhwa Pharmaceutical Co., Ltd.), whereas those in Group F received fentanyl 1 μg/kg (2 mL: 0.1 mg; Yichang Humanwell Pharmaceutical Co., Ltd.). One minute later, an initial dose of propofol was administered according to the sequential allocation schedule and injected slowly at approximately 0.5 mL/s. The prespecified target sedation level for endoscope insertion was deep sedation, defined as a Modified Observer’s Assessment of Alertness/Sedation (MOAA/S) score ≤ 1, indicating no response to mild prodding or shaking and either a response only to trapezius squeeze or no response even to trapezius squeeze. Endoscope insertion was initiated only after this target level had been achieved. During scope insertion, the anesthesiologist gently elevated the mandible to improve oral exposure and facilitate scope passage. All procedures were performed by a chief endoscopist with more than 10 years of experience in upper gastrointestinal endoscopy, using routine standardized insertion techniques at our institution. Sedation depth and insertion-related responses were continuously assessed by an experienced anesthesiologist throughout the procedure. Supplemental propofol boluses (0.5 mg/kg) were administered as needed to maintain adequate procedural sedation (MOAA/S < 3) and suppress insertion-related responses.

Upon completion of the examination, patients were transferred to the post-anesthesia care unit (PACU), where their recovery of consciousness was assessed every minute by anesthesia nurses using verbal stimuli and gentle tactile stimulation until spontaneous eye opening was observed. Discharge from the PACU was permitted once patients achieved three consecutive Modified Aldrete scores of ≥9 (11).

Non-invasive blood pressure (NIBP) was measured at 2-min intervals during endoscopy. If systolic blood pressure (SBP) fell below 80 mmHg or mean arterial pressure (MAP) dropped below 60 mmHg, intravenous ephedrine (3–12 mg) was administered as a safety intervention. Bradycardia (HR < 50 beats/min) was treated with intravenous atropine (0.3–0.5 mg). If SpO2 decreased to <95% for more than 1 min or to <90% at any time, airway interventions (jaw lift, oxygen flow augmentation) were instituted. If these measures were insufficient, the procedure was interrupted, and either an oral or nasal airway was inserted, or endotracheal intubation was performed to secure ventilation.

### Biased coin up-and-down sequential dose-finding design

(1) Dose transition rules of biased coin up-and-down design

The first patient received the starting propofol dose, and the dose for each subsequent patient was determined according to the response of the previous patient during gastroscope insertion. If, 1 min after drug administration, the patient’s MOAA/S score remained >1 or signs of inadequate sedation (such as furrowing of the brow, tearing, coughing, hiccups, or body movement) occurred during insertion, the response was classified as positive, indicating sedation failure. In such cases, an additional propofol bolus of 0.5 mg/kg was administered, and the endoscopic procedure was continued after the target sedation depth had been achieved. For the next patient, the propofol dose was increased. Conversely, if the MOAA/S score was ≤1 and no insertion-related reaction occurred, the response was classified as negative, indicating successful suppression of the insertion response. In that case, the dose for the next patient was determined using biased coin randomization, with an 11% probability of dose reduction and an 89% probability of maintaining the same dose. This randomization was implemented using computer-generated random numbers between 1 and 9, where a result of 1 indicated dose reduction and all other results indicated no change.

(2) Dose spacing and boundaries

Previous studies have reported that the ED_50_ of propofol for suppressing upper gastrointestinal endoscope insertion responses was 1.632 mg/kg when combined with 0.1 mg/kg nalbuphine (12). In contrast, the ED50 and ED95 of propofol alone for the same endpoint were 1.90 and 2.15 mg/kg, respectively (13). On the basis of these data, the effective dose of propofol for suppressing insertion responses in the present study was expected to lie within the range of 1.6–2.2 mg/kg. Accordingly, four dose levels–1.6, 1.8, 2.0, and 2.2 mg/kg–were selected to balance the risk of sedation failure at lower doses against the potential for dose-related adverse events at higher doses, while allowing adequate characterization of the dose-response profile.

### Study outcomes

The primary outcome of the study was to determine the ED_90_ of propofol, combined with either oliceridine or fentanyl, required to suppress upper gastrointestinal endoscopy insertion responses.

Secondary outcomes included the incidence of oxygen saturation and blood pressure events recorded using predefined observational thresholds. Oxygen saturation events were recorded as SpO2 < 95% and SpO2 < 90%, and blood pressure events included SBP < 90 mmHg and SBP < 80 mmHg. Additional hemodynamic outcomes included the incidence of bradycardia (HR < 50 beats/min) and the use and dosage of vasoactive drugs. Changes in MAP and HR were assessed at predefined time points (T0: admission before induction; T1: MOAA/S ≤ 1; T2: start of gastroscopy; T3: eye opening during recovery). Procedural outcomes included endoscopic procedure duration (defined as the time from endoscope insertion to removal), sedation duration (defined as the time from anesthetic administration to eye opening), as well as total propofol consumption. Other secondary outcomes included the incidence of injection pain, hiccups, and postoperative adverse events [including postoperative agitation, postoperative nausea and vomiting (PONV), and dizziness]. Fatigue at discharge was assessed using a numerical rating scale (0–10).

### Sample size

Currently, there is no standardized formula for sample size calculation in the Up-and-Down sequential design, and sample size determination is typically based on empirical rules and simulation results. Previous studies suggest that at least 40 patients are required for stable results in biased coin sequential designs. However, recent research indicates that a sample size of 50–60 patients per group provides more reliable and stable estimates (14). Considering potential dropout rates and other influencing factors, this study decided to enroll 60 patients per group, totaling 120 patients for observation and analysis.

### Statistical methods

Data analysis and graphing were performed using SPSS 26.0 and GraphPad 8.0. Normally distributed quantitative data are presented as mean ± standard deviation (SD), and group comparisons were performed using the independent samples *t*-test. Non-normally distributed data are expressed as median (M) and interquartile range (IQR), with comparisons between groups conducted using the Mann-Whitney U test. Categorical data are presented as count (%) and compared using the Chi-square test or Fisher’s exact test. A two-tailed test was used, and *P* < 0.05 was considered statistically significant.

The ED_90_ and its 95% confidence interval (CI) were estimated using Centered Isotonic Regression (CIR) and the Bootstrap method (2000 repetitions) with R software (version 3.4.4). For repeated measures data, repeated measures analysis of variance (ANOVA) was employed, with Greenhouse-Geisser correction applied if sphericity test was not met. Comparisons of ED_90_ were made using the Mann-Whitney U test and the confidence interval method. A difference was considered statistically significant if *P* < 0.05 or if the confidence intervals did not overlap.

## Results

### Characteristics of patients

**FIGURE 1:**
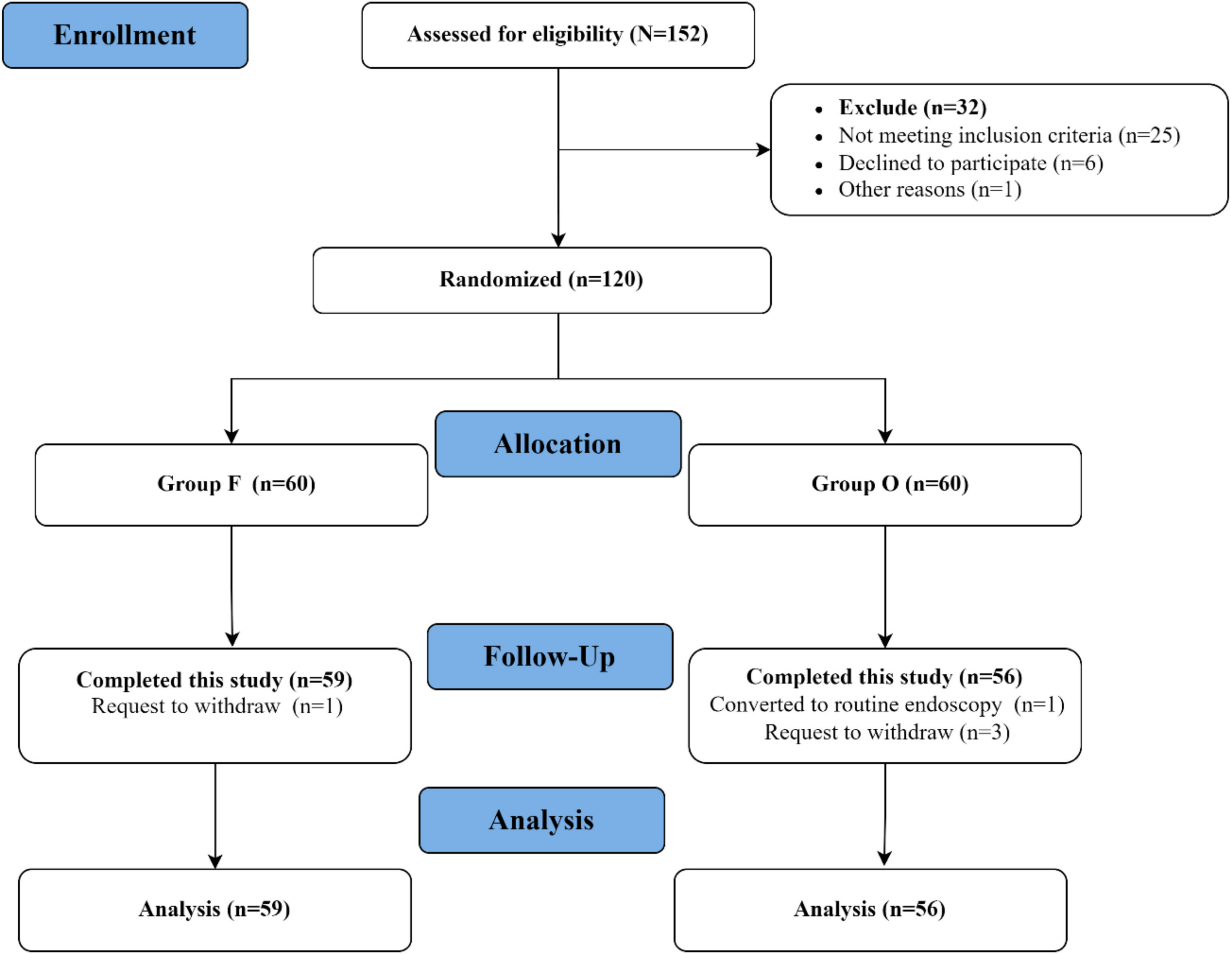
Flow diagram of participant inclusion.

A total of 152 patients were assessed for eligibility during the study period, and 120 were enrolled after meeting the inclusion criteria. Five patients were excluded from the final analysis owing to voluntary withdrawal or conversion to routine gastroscopy, leaving 115 patients for analysis, including 59 in Group F and 56 in Group O (Figure 1). The two groups were comparable with respect to baseline demographic and clinical characteristics, including age and sex distribution (*P* > 0.05; Table 1).

**TABLE 1:** Demographic characteristics and clinical variables.

Variable	Group F (*N* = 59)	Group O (*N* = 56)	*P*
Age (years)	49.6 ± 11.9	48.9 ± 11.0	0.774
**Sex**			0.113
Male	34 (57.6)	24 (42.9)	
Female	25 (42.4)	32 (57.1)	
BMI (kg/m^2^)	23.5 ± 2.7	23.6 ± 3.3	0.849
**ASA physical status**			0.526
I	8 (13.6)	10 (17.9)	
II	51 (86.4)	46 (82.1)	
Hypertension	12 (20.3)	16 (28.6)	0.304
Endoscopic procedure duration (min)	4.2 ± 1.3	4.4 ± 1.6	0.052
Sedation duration (min)	7.5 ± 1.3	7.2 ± 1.7	0.141
Total propofol dose (mg)	130.3 ± 21.0	125.9 ± 24.2	0.140

### Dose-response

Table 2 summarizes the response to gastroscopy insertion at different propofol dose levels in both groups, including the total number of cases, the number of successful cases, success rate, and success rate adjusted by the PAVA algorithm, as shown in Table 2. Using the centered isotonic regression (CIR) method, the estimated ED_90_ of propofol was 1.942 mg/kg (95% CI: 1.709–2.494 mg/kg) in Group F and 1.871 mg/kg (95% CI: 1.620–2.519 mg/kg) in Group O. Although the ED90 estimate was numerically lower in Group O, the difference between groups was not statistically significant (*z* = −0.693, *P* = 0.488).

**TABLE 2:** Observed response rates and PAVA-adjusted rates for propofol using the centered isotonic regression method in Group F and Group O.

Group	Dose-level (mg/kg)	Number of cases, *n*	Successful cases, *n*	Observed success rate	PAVA-adjusted success rate
Group F					
	1.6	15	12	0.8	0.806
	1.8	26	22	0.846	0.848
	2	13	12	0.923	0.921
	2.2	5	5	1	0.983
Group O					
	1.6	11	9	0.818	0.825
	1.8	25	22	0.88	0.881
	2	16	15	0.938	0.935
	2.2	4	4	1	0.988

**FIGURE 2:**
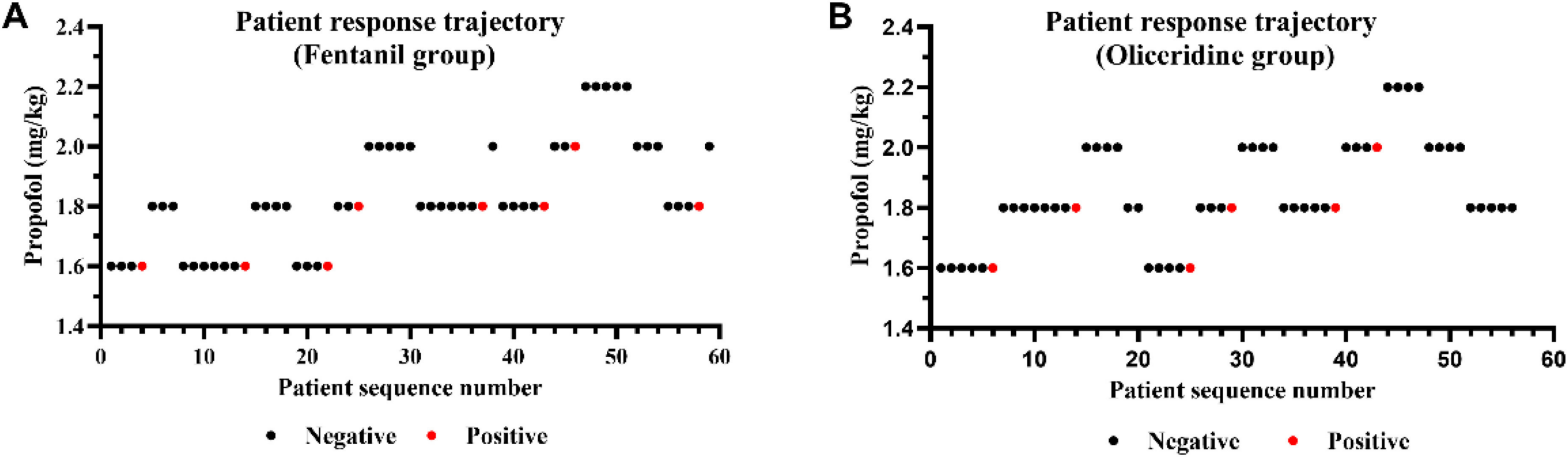
Experimental trajectories of patient responses in the fentanyl group (Group F) and oliceridine group (Group O). **(A)** Patient response trajectory in Group F. **(B)** Patient response trajectory in Group O. Black dots indicate negative responses (successful suppression), and red dots indicate positive responses (sedation failure).

Figures 2A,B present the sequential allocation plots for Group F and Group O, respectively. The *x*-axis represents patient sequence, and the *y*-axis indicates the administered propofol dose level. Successful and failed insertion responses are indicated by black and red circles, respectively.

**FIGURE 3:**
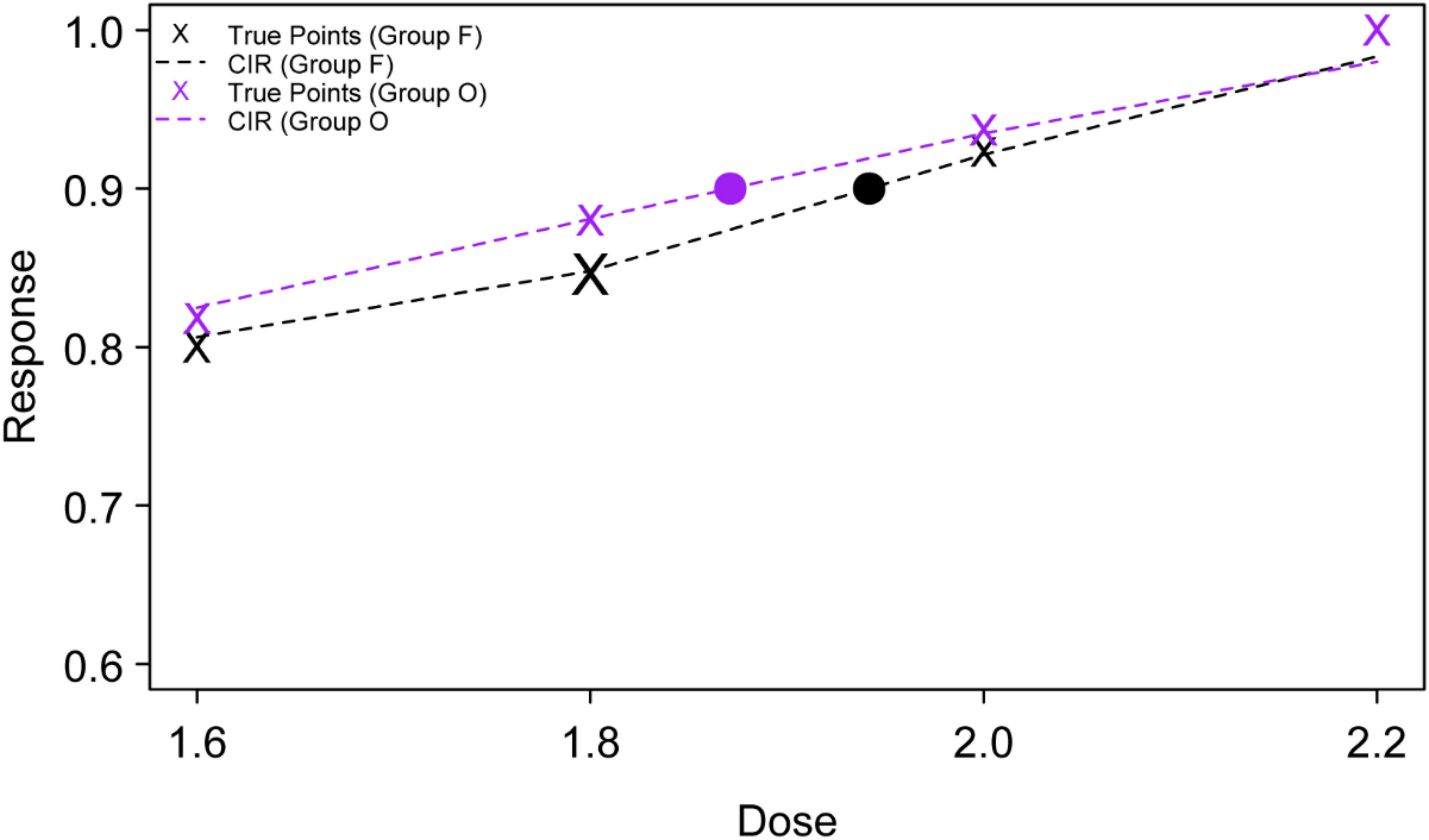
Dose-response curves for Group F and Group O estimated using centered isotonic regression (CIR).

Figure 3 shows the dose-response curves fitted using the CIR method. The *x*-axis represents the propofol dose (mg/kg), and the *y*-axis represents the probability of successful insertion. The “×” symbols indicate the observed success rates at each dose level, while the dashed curves represent the fitted dose-response relationships for Group O and Group F. The corresponding estimated ED_90_ values are indicated by filled markers.

### MAP and HR change over time

**FIGURE 4:**
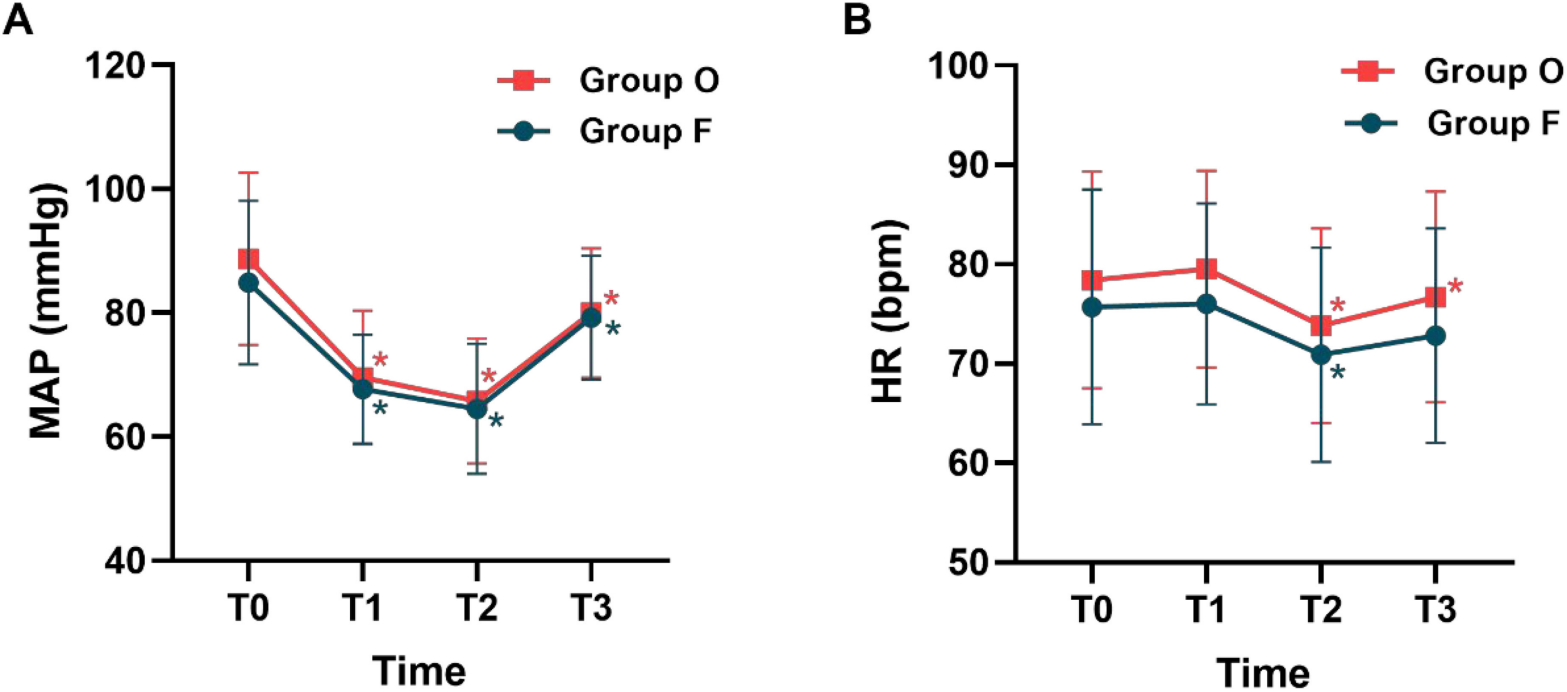
Changes in MAP **(A)** and HR **(B)** over time. Red squares indicate Group F, and blue circles indicate Group O; T0, admission before induction; T1, MOAA/S ≤ 1; T2, start of gastroscopy; T3, eye opening during recovery. *P < 0.05 compared with T0.

Repeated-measures analysis of variance showed no significant interaction between group and time for either MAP or HR (both *P* > 0.05). Accordingly, main effect analyses were performed. The main effect of group was not significant for MAP (*F* = 1.464, *P* = 0.229) or HR (*F* = 3.500, *P* = 0.064), indicating no overall differences between Group F and Group O across time points. In contrast, a significant main effect of time was observed for both MAP and HR (both *P* < 0.05), demonstrating significant changes in these variables over the course of the procedure. *Post hoc* comparisons under the time main effect revealed that, compared with T0, MAP was significantly reduced from T1 to T3 in both groups (*P* < 0.05). HR was significantly decreased at T2 and T3 in Group F, whereas in Group O a significant decrease in HR was observed only at T2 (*P* < 0.05) (Figure 4).

### Adverse effects

The incidence of oxygen desaturation to SpO2 < 95% and SpO2 < 90%, the need for airway intervention, hypotension requiring vasoactive treatment, NRS fatigue score ≥ 4, dizziness, and PONV were numerically lower in Group O than in Group F; however, these differences did not reach statistical significance (*P* > 0.05). No significant between-group differences were found for other outcomes (*P* > 0.05). Additionally, no patients experienced postoperative agitation, severe PONV, or other serious adverse events during the study. Detailed information is provided in Table 3.

**TABLE 3:** Adverse effects.

Outcome	Group F (*N* = 59)	Group O (*N* = 56)	*P*
SpO2 < 95%	8 (13.6)	4 (7.1)	0.246
90% ≤ SpO2 < 95%	4 (6.8)	3 (5.4)	
SpO2 < 90%	4 (6.8)	1 (1.8)	
Requirement for airway intervention	7 (11.9)	4 (7.1)	0.390
SBP < 90 mmHg	35 (59.3)	34 (60.7)	0.818
80 mmHg ≤ SBP < 90 mmHg	18 (30.5)	21 (37.5)	
SBP < 80 mmHg	17 (28.8)	13 (23.2)	
Hypotension requiring treatment	23 (39.0)	21 (37.5)	0.870
Ephedrine (mg), Median (IQR)	0 (0, 6)	0 (0, 6)	0.725
Bradycardia	1 (1.7)	0 (0)	0.328
Injection pain	6 (10.2)	5 (8.9)	0.821
Hiccups	3 (5.1)	4 (7.1)	0.645
NRS fatigue score, Median (IQR)	1 (1, 2)	1 (1, 2)	0.978
NRS fatigue score ≥ 4	3 (5.1)	1 (1.8)	0.335
Dizziness in PACU	8 (13.6)	7 (12.5)	0.866
PONV in PACU	4 (6.8)	3 (5.4)	0.750

## Discussion

This randomized dose-finding study estimated the ED_90_ of propofol for suppressing gastroscopy insertion responses when combined with fentanyl or oliceridine using a biased coin up-and-down sequential design. The estimated ED_90_ values were 1.942 mg/kg (95% CI, 1.709–2.494) for fentanyl and 1.871 mg/kg (95% CI, 1.620–2.519) for oliceridine. These values were not significantly different, indicating similar propofol requirements for the two opioid adjuncts under the study conditions. These findings provide quantitative reference data for individualized initial propofol dosing and support comparable propofol requirements for fentanyl and oliceridine under the study conditions.

90% effective dose has particular practical relevance in procedural sedation because it lies near the inflection region of the sigmoid dose-response curve and represents a high-probability effective dose (15, 16). When the administered dose falls below this level, the probability of success decreases steeply, whereas dose escalation beyond this range is associated with only marginal additional improvement in success rate. From a clinical perspective, anesthesiologists generally seek an initial dose with a high probability of achieving adequate sedation and favorable insertion conditions, rather than one that is effective in only 50% of patients (17). Accordingly, ED_90_ is more clinically useful than ED_50_ as a reference for initial dosing in gastroscopy, where insufficient suppression of gagging, coughing, retching, or body movement may adversely affect both patient comfort and procedural quality.

In routine clinical practice, propofol dosing during upper gastrointestinal endoscopy is usually individualized and titrated according to patient response (18). However, the absence of standardized quantitative dose-response benchmarks makes it difficult to objectively compare propofol requirements across different opioid adjuncts. Estimating a high-probability effective dose therefore provides a reproducible pharmacodynamic reference for defining propofol requirements under controlled conditions (19, 20). Previous studies have shown that the ED_50_ of propofol alone for suppressing gastroscope insertion responses is approximately 1.90 mg/kg (13). In the present study, the estimated ED_90_ of propofol was 1.942 mg/kg when combined with fentanyl and 1.871 mg/kg when combined with oliceridine, values that were numerically close to the reported ED_50_ of propofol alone. Although this cross-study comparison should be interpreted with caution, the numerical proximity between the present ED_90_ estimates and the previously reported ED_50_ of propofol alone raises the possibility that adjunct opioid administration may improve suppression of the insertion response at a given propofol dose. In other words, when fentanyl or oliceridine is co-administered, a given dose of propofol may be more likely to achieve successful insertion conditions than the same dose of propofol used alone. Clinically, such a pharmacodynamic interaction may reduce the need for further propofol dose escalation, which is important because higher propofol doses are associated with an increased risk of respiratory depression and hemodynamic instability.

Fentanyl is among the most widely used opioid adjuncts in propofol-based sedation for gastrointestinal endoscopy and therefore serves as a clinically meaningful reference comparator (21). Oliceridine, a newer μ-opioid receptor agonist with G protein-biased signaling properties, has emerged as a potential alternative opioid adjunct, with the potential advantage of a lower incidence of opioid-related adverse events (22, 23). Previous studies have suggested that oliceridine has an analgesic potency approximately five times that of morphine, whereas fentanyl is estimated to be about 100 times more potent than morphine (24, 25). Accordingly, the study doses of oliceridine 20 μg/kg and fentanyl 1 μg/kg were considered broadly equianalgesic. In the present study, the estimated ED_90_ of propofol was numerically lower in the oliceridine group, but the between-group difference was not statistically significant. Taken together, these results suggest that, within the dosing framework tested here, oliceridine and fentanyl provided broadly comparable adjunctive effects on propofol-mediated suppression of gastroscope insertion responses.

Recent studies have raised the possibility that oliceridine may offer a more favorable safety profile than conventional opioids in procedural sedation (26, 27). In our study, the incidences of oxygen desaturation, airway intervention, and hypotension requiring treatment were also numerically lower in the oliceridine group, although these differences were not statistically significant. Several factors may explain this finding. Most importantly, this study was powered for dose estimation rather than for comparative safety analysis. In addition, the sequential dose-finding design, with stepwise adjustment of propofol dose according to the response of the preceding patient, may have influenced the occurrence and distribution of adverse events. Therefore, although the observed numerical trends may be of clinical interest, they should be regarded as exploratory and require confirmation in adequately powered confirmatory studies.

It should be noted that the present ED_90_ estimates reflect not only the pharmacologic effects of the study drugs, but also the specific procedural and methodological conditions under which they were obtained. Previous studies have shown that suppression of gastroscope insertion responses is influenced by factors beyond the sedative regimen itself, including endpoint definition, sedation strategy, and the intensity of procedural stimulation (28–30). In clinical practice, the targeted depth of sedation, the timing of adjunct opioid administration, the rate of propofol injection, and operator technique may all affect the occurrence of gagging, coughing, retching, or body movement at insertion (31–33). Although we attempted to reduce such variability through a predefined sedation target, fixed opioid doses, a standardized propofol administration protocol, and a single experienced endoscopist, these influences cannot be fully eliminated. Therefore, the present dose estimates should be regarded as evidence-based reference values for initial clinical dosing rather than fixed dosing standards, and should be further titrated according to individual patient characteristics, procedural conditions, and real-time responses.

Several limitations of this study should be acknowledged. First, as a biased coin up-and-down dose-finding trial, the sample size was designed for ED_90_ estimation rather than for detecting between-group differences in safety or other clinical outcomes; therefore, analyses of adverse events and other secondary endpoints should be interpreted as exploratory. Second, this was a single-center study conducted in relatively healthy adults, which may limit the generalizability of the findings to older, obese, or higher-risk patients, particularly those with significant cardiopulmonary comorbidities. Third, although the target sedation depth was predefined and assessed using the MOAA/S scale, sedation depth was evaluated clinically rather than with objective electroencephalographic monitoring, such as bispectral index monitoring, and some degree of assessment variability cannot be excluded. Additionally, although the inclusion criteria limited the ranges of age and BMI, insertion responses may still differ according to sex, age, and body weight. Thus, the present study should be regarded as a preliminary dose-finding investigation, and further studies are warranted to establish optimal dosing in different patient subgroups. Future studies should include multicenter validation in broader and higher-risk populations, evaluate age- and sex-specific dosing characteristics, compare different opioid doses and sedation strategies, and use adequately powered confirmatory designs to assess comparative safety and clinical outcomes.

## Conclusion

In conclusion, the ED_90_ of propofol for suppressing gastroscopy insertion responses was 1.942 mg/kg (95% CI, 1.709–2.494) when combined with fentanyl 1 μg/kg and 1.871 mg/kg (95% CI, 1.620–2.519) when combined with oliceridine 20 μg/kg. The substantial overlap of the confidence intervals suggests comparable propofol requirements for the two opioid adjuncts under the study conditions. In patients similar to those included in this study, an initial propofol dose of approximately 1.9 mg/kg may serve as a practical reference when either fentanyl or oliceridine is used as an adjunct, although dosing should remain individualized and further validation is needed in broader patient populations and alternative sedation settings.

## Plain language summary

During upper gastrointestinal endoscopy, propofol is commonly used to provide sedation, but it can cause breathing and blood pressure problems, especially at higher doses. To improve sedation quality and reduce the required dose of propofol, opioids such as fentanyl are often added. Oliceridine is a newer opioid that may offer similar pain relief with fewer side effects. In this study, we aimed to determine the dose of propofol required to successfully suppress the insertion response during gastroscopy when combined with either fentanyl or oliceridine. Using a dose-finding study design, we estimated the dose of propofol that was effective in 90% of patients. We found that similar doses of propofol were required when combined with fentanyl or oliceridine to achieve successful sedation. This suggests that, within the conditions of this study, both drug combinations provided comparable effectiveness for suppressing the gastroscopy insertion response.

## Data Availability

The raw data supporting the conclusions of this article will be made available by the authors, without undue reservation.

## References

[1] Attia A Yeluri S Samuel N Balchandra S Vasas P. Intra-Operative upper GI endoscopy helps to identify the gastro-jejunostomy perforation site in Roux-en-Y gastric bypass patient. *Obes Surg.* (2024) 34:1993–4. 10.1007/s11695-024-07202-8 38564176

[2] Yang W Zhou R Zhou X Chen X Zhou D Zhang X. Effective dose of oliceridine fumarate co-administered with remimazolam in suppressing gastroscope insertion responses for adults. *Drug Des Devel Ther.* (2025) 19:5033–41. 10.2147/DDDT.S527586 40524805 PMC12169015

[3] Sahinovic MM Struys M Absalom AR. Clinical pharmacokinetics and pharmacodynamics of propofol. *Clin Pharmacokinet.* (2018) 57:1539–58. 10.1007/s40262-018-0672-3 30019172 PMC6267518

[4] Zheng Y Xu Y Huang B Mai Y Zhang Y Zhang Z. Effective dose of propofol combined with a low-dose esketamine for gastroscopy in elderly patients: a dose finding study using dixon’s up-and-down method. *Front Pharmacol.* (2022) 13:956392. 10.3389/fphar.2022.956392 36204220 PMC9530901

[5] Yin N Xia J Cao YZ Lu X Yuan J Xie J. Effect of propofol combined with opioids on cough reflex suppression in gastroscopy: study protocol for a double-blind randomized controlled trial. *BMJ Open.* (2017) 7:e014881. 10.1136/bmjopen-2016-014881 28864688 PMC5589021

[6] Doğanay G Ekmekçi P Kazbek BK Yılmaz H Erkan G Tüzüner F et al. Effects of alfentanil or fentanyl added to propofol for sedation in colonoscopy on cognitive functions: randomized controlled trial. *Turk J Gastroenterol.* (2017) 28:453–9. 10.5152/tjg.2017.16489 28928100

[7] Gu Z Xin L Wang H Hu C Wang Z Lu S et al. Doxapram alleviates low SpO2 induced by the combination of propofol and fentanyl during painless gastrointestinal endoscopy. *BMC Anesthesiol.* (2019) 19:216. 10.1186/s12871-019-0860-1 31757206 PMC6873474

[8] Su M Zhu Y Liu S Song L Qu J Zhang Y et al. Median effective dose (Ed(50)) of esketamine combined with propofol for children to inhibit response of gastroscope insertion. *BMC Anesthesiol.* (2023) 23:240. 10.1186/s12871-023-02204-y 37464290 PMC10354894

[9] Markham A. Oliceridine: first approval. *Drugs.* (2020) 80:1739–44. 10.1007/s40265-020-01414-9 33025536

[10] Ma B Li Y Leng C Ji A Zhang N Tao X et al. A comparative evaluation of the safety and efficacy of oliceridine and sufentanil in gastrointestinal endoscopy: a single-center. *Randomized Controlled Trial. Drug Des Devel Ther.* (2025) 19:5111–21. 10.2147/DDDT.S512529 40546662 PMC12182078

[11] Ding D Ishag S. *Aldrete Scoring System.* Treasure Island, FL: Statpearls (2023). 37603628

[12] Tang L Ye C Wang N Chen C Chen S Gao S et al. The median effective doses of propofol combined with two different doses of nalbuphine for adult patients during painless gastroscopy. *Front Pharmacol.* (2022) 13:1014486. 10.3389/fphar.2022.1014486 36204238 PMC9531776

[13] Liu FK Wan L Shao LJZ Zou Y Liu SH Xue FS. Estimation of effective dose of propofol mono-sedation for successful insertion of upper gastrointestinal endoscope in healthy, non-obese Chinese adults. *J Clin Pharm Ther.* (2021) 46:484–91. 10.1111/jcpt.13312 33217028

[14] Oron AP Souter MJ Flournoy N. Understanding research methods: up-and-down designs for dose-finding. *Anesthesiology.* (2022) 137:137–50. 10.1097/ALN.0000000000004282 35819863

[15] Du J Li C Huang M Qin X. Comparison of the Effective Dose 90 (Ed90) and clinical outcomes of fentanyl versus esketamine for analgesia in hysteroscopy: a two-part, randomized, double-blind trial. *Drug Des Devel Ther.* (2026) 20:574516. 10.2147/DDDT.S574516 41858911 PMC12998388

[16] Li S Pan X Zhu Y Liu J Li Z Gao X et al. The 90% minimum effective dose of oxycodone for thoracoscopic lobectomy in elderly patients: a double-blind study using a biased-coin design. *Drug Des Devel Ther.* (2026) 20:560051. 10.2147/DDDT.S560051 41743213 PMC12929209

[17] Guntz E Latrech B Tsiberidis C Gouwy J Kapessidou Y. ED50 and ED90 of intrathecal hyperbaric 2% prilocaine in ambulatory knee arthroscopy. *Can J Anaesth.* (2014) 61:801–7. 10.1007/s12630-014-0189-7 24906303

[18] Yu J Xiang B Song Y Chen H Li Y Liu C. ED50 of propofol in combination with low-dose sufentanil for intravenous anaesthesia in hysteroscopy. *Basic Clin Pharmacol Toxicol.* (2019) 125:460–5. 10.1111/bcpt.13280 31231918

[19] Lu Z Zhou N Li Y Yang L Hao W. Up-down determination of the 90% effective dose (ED90) of remimazolam besylate for anesthesia induction. *Ann Palliat Med.* (2022) 11:568–73. 10.21037/apm-22-89 35249335

[20] Silva MPD Matsui C Kim DD Vieira JE Malheiros CA Mathias LAST. Sugammadex ED90 dose to reverse the rocuronium neuromuscular blockade in obese patients. *Rev Col Bras Cir.* (2017) 44:41–5. 10.1590/0100-69912017001010 28489210

[21] Inam K Qazi MS Ahmed M Hassan A Yaseen I Khan MA et al. Efficacy and safety of ketamine or esketamine versus fentanyl-class opioids as adjuncts to propofol for gastrointestinal endoscopy: a systematic review and meta-analysis. *A A Pract.* (2026) 20:e02177. 10.1213/XAA.0000000000002177 41955406

[22] Ni Y Huang R Yang S Yang XY Zeng S Yao A et al. Pharmacokinetics and safety of oliceridine fumarate injection in chinese patients with chronic non-cancer pain: a Phase I, single-ascending-dose, open-label clinical trial. *Drug Des Devel Ther.* (2024) 18:2729–43. 10.2147/DDDT.S461416 38974123 PMC11227858

[23] Simons P van der Schrier R van Lemmen M Jansen S Kuijpers KWK van Velzen M et al. Respiratory effects of biased ligand oliceridine in older volunteers: a pharmacokinetic-pharmacodynamic comparison with morphine. *Anesthesiology.* (2023) 138:249–63. 10.1097/ALN.0000000000004473 36538359

[24] Yi K Sun W Yu W Chen S. Overview and prospects of the clinical application of oliceridine. *Drug Des Devel Ther.* (2025) 19:5415–30. 10.2147/DDDT.S525471 40599606 PMC12209531

[25] Senguttuvan NB Suman F Paneerselvam T Malepati B Ramesh S Vallivedu MV et al. Comparison of the effect of morphine and fentanyl in patients with acute coronary syndrome receiving ticagrelor - The COMET (Comparison Morphine, Fentayl and Ticagrelor) randomized controlled trial. *Int J Cardiol.* (2021) 330:1–6. 10.1016/j.ijcard.2021.02.037 33600846

[26] Chen L Xie K Ji K Long M Zhang Y He K. Effects of oliceridine versus sufentanil on hemodynamic stability in elderly hypertensive patients during laryngeal mask airway anesthesia: a randomized controlled trial. *Drug Des Devel Ther.* (2025) 19:9515–22. 10.2147/DDDT.S547901 41147005 PMC12554289

[27] Ke Z He Y Hu Q Zheng D Yao Z Zhou W. A comparison of the effects of oliceridine and sufentanil on the quality of recovery after hysteroscopic surgery: a prospective double-blind randomized controlled trial. *J Anesth.* (2026) 40:235–44. 10.1007/s00540-025-03578-8 40946263

[28] Ahn H Chae YJ Choi GB Lee MG Yoo JY. Determining the optimal dosage of dexmedetomidine for smooth emergence in older patients undergoing spinal surgery: a study of 44 Cases. *Med Sci Monit.* (2024) 30:e944427. 10.12659/MSM.944427 38851875 PMC11171429

[29] Kim HI Jung DH Lee SJ Kim N Kim SH Ji YJ et al. Determining ED90 of flumazenil for selective respiratory distress improvement using remimazolam during endoscopic submucosal dissection of gastric neoplasms: a prospective study. *Cancers.* (2025) 17:321. 10.3390/cancers17020321 39858103 PMC11763922

[30] Xu Q Qian J Zhang SQ Xia F Hu HJ Xiao F. Dose-Response study of remimazolam combined with remifentanil for attenuating stress response during laryngeal mask airway insertion in elderly female patients: a prospective double-blinded study. *Drug Des Devel Ther.* (2025) 19:1575–83. 10.2147/DDDT.S494426 40066083 PMC11891449

[31] Sun H Wang T Xu ZX Chen XF Cao JB Li H. [Effective dose and adverse reactions analysis of remimazolam for sedation in elderly patients undergoing gastroscopy]. *Zhonghua Yi Xue Za Zhi.* (2022) 102:332–5. 10.3760/cma.j.cn112137-20211111-02509 35092973

[32] Zhao J Zhang Y Su G Wang S Zhang X Wang G et al. The median effective dose of ciprofol combined with a low-dose sufentanil for gastroscopy in obese or nonobese patients: a dose-finding study using Dixon’s up-and-down method. *Front Pharmacol.* (2025) 16:1521715. 10.3389/fphar.2025.1521715 40041487 PMC11876871

[33] Zhao L Zhou X Chen L Mao W Guo Y Liu X et al. The 50% effective dose of remimazolam combined with different doses of esketamine for painless gastroscopy. *Sci Rep.* (2025) 15:12770. 10.1038/s41598-025-97649-1 40229355 PMC11997078

